# Effects of multiple vitamin E levels and two fat sources in diets for swine fed to heavy slaughter weight of 150 kg: II. Tissue fatty acid profile, vitamin E concentrations, immune capacity, and antioxidant capacity of plasma and tissue

**DOI:** 10.1093/tas/txad087

**Published:** 2023-07-28

**Authors:** Ding Wang, Young Dal Jang, Marlee Kelley, Gregg K Rentfrow, Michael J Azain, Merlin D Lindemann

**Affiliations:** University of Kentucky, Department of Animal and Food Sciences, Lexington, KY 40546, USA; University of Georgia, Department of Animal and Dairy Sciences, Athens, GA 30602, USA; University of Kentucky, Department of Animal and Food Sciences, Lexington, KY 40546, USA; University of Kentucky, Department of Animal and Food Sciences, Lexington, KY 40546, USA; University of Georgia, Department of Animal and Dairy Sciences, Athens, GA 30602, USA; University of Kentucky, Department of Animal and Food Sciences, Lexington, KY 40546, USA

**Keywords:** fat, fatty acid profile, heavy slaughter weight pigs, tocopherol concentration, vitamin E level

## Abstract

The study objective was to evaluate the effect of two fat sources and graded levels of vitamin E (**VE**) supplementation on tissue fatty acid profile, VE concentrations, immune capacity, and antioxidant capacity of plasma and tissues of pigs at heavy slaughter weight (150 kg). A total of 48 individually-fed pigs (24 barrows, 24 gilts; 28.44 ± 2.69 kg) were randomly assigned to eight dietary treatments in a 2 × 4 factorial arrangement. The two fat treatments were either 5% tallow (**TW**) or 5% distiller’s corn-oil (**DCO**). The VE treatments included four levels of α-tocopheryl-acetate (11, 40, 100, and 200 ppm). Compared to pigs fed the DCO diet, pigs fed the TW diet had greater SFA (C14, C16, and C18; *P* < 0.05) and MUFAs (C14:1, C16:1, C18:1, and C20:1; *P* < 0.05), lower PUFA (C18:2n-6, C18:3n-3, C20:2, C20:3, and C20:4; *P* < 0.05) and iodine value in the backfat and belly fat. Increasing dietary VE supplementation level increased α- and total tocopherol concentrations in plasma (linear and quadratic, *P* < 0.05), liver, and loin muscle (linear, *P* < 0.06), superoxide dismutase activity (quadratic, *P* < 0.05), but decreased γ-tocopherol concentrations in liver (linear, *P* = 0.06), plasma, and loin muscle (quadratic, *P* < 0.07), and decreased liver glutathione disulfide (**GSSG**; linear, *P* = 0.07) and malondialdehyde (**MDA**) content (quadratic, *P* < 0.05). There was an interaction between fat sources and dietary VE supplementation level on the concentration of α-tocopherol in the loin muscle (*P* < 0.05) wherein a greater increase was observed in the TW treatment than the DCO treatment with the increasing dietary VE supplementation level. In conclusion, dietary FA composition in TW and DCO affected the composition of most FA in backfat, belly fat, and liver while increasing VE supplementation level did not significantly alter the FA profile in these tissues. Increasing dietary VE supplementation level increased tocopherol concentrations in plasma, liver and loin muscle, and improved antioxidant capacity while tocopherol concentrations in plasma, liver and loin muscle in the TW treatment increased more than they did in the DCO treatment.

## Teaser

1.Increasing Vitamin E supplementation increases tissue content of Vitamin E but has little impact on fatty acid content of tissues.

2.Different fat sources clearly affect the fatty acid content of tissues and have differing effects on plasma Vitamin E content.

## Introduction

Global pork consumption has increased continually in the past decades; the increased demand for pork has been met by an increase in both the number of pigs produced and their slaughter weight (**SLW**). However, there has been less research on the growth performance and carcass traits of pigs raised to heavy slaughter weights up to 150 kg. Supplemental fat is a primary energy source and one of the most important factors that determines the fatty acid (**FA**) composition of pork. Each supplemental fat source has a unique FA profile (e.g., carbon chain length, degree of unsaturation, and position of the double bonds) and, thus, affect pork quality differently (de Tonnac and Mourot, 2018). It has been demonstrated that the loin of pigs fed diets containing 5% soybean oil had greater polyunsaturated FA (**PUFA**) percentages and iodine values (**IV**) than those fed no additional fat or fed diets containing 5% beef tallow and poultry fat (Apple et al., 2009b).

Vitamin E (**VE**) is an essential micronutrient for pigs and plays important role in reducing oxidative stress as an antioxidant (Boler et al., 2009). In our previous studies (Wang et al., 2022a, 2022b), VE supplementation at the high level of 200 IU/kg diet increased plasma and tissue VE concentrations but did not alter FA composition in adipose tissues and liver while FA composition of the supplemental fat sources, and thereby diets, affected plasma VE concentrations; agreeing with others that VE absorption and bioavailability depends on both the dose and the duration of dosing (Blatt et al., 2004) as well as the properties of the dietary fat (Prévéraud et al., 2014). Previously, Trefan et al. (2011) suggested that at least 100 IU VE/kg diet was required to significantly improve lipid oxidation protection of longissimus muscle, which is much greater than the NRC (2012) VE requirement estimate (11 IU/kg). Additionally, a few studies have investigated supplementation levels of VE in swine diets equal to or greater than 500 IU/kg (Jensen et al., 1997; Onibi et al., 2000; Hasty et al., 2002). However, while a high level of VE supplementation can increase plasma and tissue (liver and loin muscle) VE concentrations and may improve feed efficiency for pigs at heavy slaughter weight, it has not yet been demonstrated how pigs respond to graded levels of vitamin E from the level of NRC requirement estimate (11 IU/kg) up to 200 IU/kg in conjunction with different fat sources. Specifically, it has not been demonstrated yet if there is any interaction between dietary fat sources and graded levels of VE supplementation when the fat sources used in the diet have a large difference in the FA composition such as tallow (**TW**), an animal fat, containing a high content of monounsaturated FA (**MUFA**) and corn oil (**CO**), a plant oil, containing a high content of polyunsaturated FA (**PUFA**).

Therefore, the objectives of this project were to identify the impacts of two dietary fat sources and graded levels of VE supplementation to pigs grown to a heavy SLW on tissue FA profile, plasma and tissue VE concentrations, and liver antioxidant capacity, and to specifically evaluate the potential interactions between dietary fat source and VE supplementation dependent on the degree of unsaturation of the dietary fat sources and the level of dietary VE.

## Materials and Methods

This experiment was carried out in an environmentally controlled room at the University of Kentucky Swine Research Center. The animal slaughter and sample collection were performed at the University of Kentucky Meat Science Laboratory. The experiment was conducted under protocols approved by the Institutional Animal Care and Use Committee of the University of Kentucky.

### Animals, Experimental Design, and Housing

A total of 48 individually fed pigs (24 barrows, 24 gilts; 28.44 ± 2.69 kg) were selected from a pool of 120 pigs, and blocked by sire, body weight (**BW**), and sex, and then randomly assigned within block to 1 of the 8 dietary treatments in a 2 × 4 factorial arrangement of fat sources and vitamin E (**VE**) levels. Fat sources included tallow (**TW**) and distiller’s corn oil (**DCO**). The four VE supplementation levels (11, 40, 100, and 200 ppm) were supplied as DL (all-rac)-α-tocopheryl acetate (**ATA**; ROVIMIX E 50 ADS, DSM Nutritional Products, Inc., Parsippany, NJ) in a dry form. As defined in NRC (2012), one IU VE is equal to 1 mg of DL-α-tocopheryl acetate. All pigs were housed in individual pens (0.61 × 2.44 m^2^) with free access to water and feed.

### Diets

The diets were corn-soybean meal based in mash form and fed to pigs with five feeding phases including 25 to 50 kg (phase 1), 50 to 75 kg (phase 2), 75 to 100 kg (phase 3), 100 to 125 kg (phase 4), and 125 to 150 kg (phase 5), respectively. All experimental diets were formulated to meet or exceed nutrient requirement estimates of NRC (2012) for growing-finishing pigs. Lysine levels for phase 4 were calculated with the formula provided by NRC (2012) because the pigs were slaughtered at ~150 kg which was greater than the final tabular weight in NRC (2012). In phase 5 (125 to 150 kg), lysine levels were chosen after consultation with industry nutritionists; the lysine level was higher in Phase 5 of the current experiment than Wang et al. (2022a) that used the level calculated with the formula provided by NRC (2012). The fat inclusion level (5%) was based on the amount of DCO that might be realized from an aggressive use of DDGS (45%) in the finishing diets. Formulas for each phase are provided in Wang et al. (2023).

Multiple batches of experimental diets were mixed for each phase. To minimize differences in non-treatment components of the diets, a basal diet for each of the two fat sources (TW and CO) was firstly mixed, to which different VE treatments including 11, 40, 100, and 200 ppm ATA were then incorporated for each experiment, respectively.

### Blood Collection

Blood samples from each pig were collected initially and at the end of each phase by vena cava puncture using a syringe and needle. After the blood collection, whole blood was transferred to a 16 × 100 mm vacutainer tube containing the anticoagulant heparin (Becton, Dickinson and Company, Franklin Lakes, NJ). Plasma samples were obtained by centrifugation at 2,500 × *g* for 20 min at 4 °C; and then aliquoted into 1.5 mL Eppendorf Safe-Lock tubes (Eppendorf North America, Hauppauge, NY), and stored at −80 °C until plasma tocopherol analysis.

### Harvest and Tissue Sampling

Pigs were slaughtered at about 150 kg live weight under USDA inspection. After being transferred to the meat lab, pigs were slaughtered after a rest of at least 30 min. The slaughter process included electrical stunning, exsanguination, dehairing, evisceration, and carcass washing. During the process of slaughter, liver and its subsamples (only left lateral lobe) were collected within 20 min after evisceration, bagged with moisture barrier bags, flash frozen with liquid nitrogen, and then stored at −80 °C for further analysis for VE concentration, antioxidants, fat content, and FA profile.

After a 24-h cooler chill, fat samples from the 10th rib backfat and the belly area even with the first rib were taken during the process of primal cut division. These samples were used for FA analysis by gas chromatography (**GC**) and the calculation IV. The fat samples were vacuum packaged, and then stored at −80 °C until transported to University of Georgia for FA profile analysis. Loin samples were also obtained after the removal of primal cuts. Two 2.54-cm chops of loin sample (around 200 g each) located at the 10th rib were collected, vacuum packaged, and stored at −22 °C until further analysis for VE concentrations.

### Chemical Analyses

Samples were analyzed for FA composition at the University of Georgia (including diets, liver, belly fat, and backfat tissues). The FA profiles were determined by **GC** using a Shimadzu gas chromatograph (Model 14 A, Columbia, MD) with a flame ionization detector the procedure was modified from Park and Goins (1994). Approximately 1 g of each fat-added diet sample, 100 mg of each adipose sample, and 2.0 g of liver were used for analysis. After thawing, the fat cores of back fat tissues were trimmed free of lean and skin and separated into the outer layer and inner layer of fat. Samples were processed through a 2-step methylation procedure. The first step was heating in 0.5 N sodium methoxide in methanol for 30 min at 90 °C, followed by the addition of boron trifluoride in methanol and heating for another 20 min. Methyl esters were then isolated in hexane and at the same time, anhydrous sodium sulfate was added to remove any residual water. The processed samples were stored at 4 °C until being analyzed. Tridecanoic acid (2 mg/mL in methanol) was used as the internal standard. Fatty acid methyl esters were separated on a Phenomenex, ZBWax Plus wide-bore capillary column (Phenomonex, Torrance, CA) with nitrogen as the carrier gas. Initial column temperature was 160 °C which was held for 10 min and increased at a rate of 5 °C/min to 220 °C. Injector temperature was 250 °C and detector temperature was 260 °C. Peaks were identified by comparison of retention times of known standards. Quantification was corrected for recovery of the internal standard and is based on the reference standard. The minimum detection limit for FAs was set at 0.01%; relative percentages of FAs detected below this level were denoted as not detected (**ND**). The IV of fat source and tissue was calculated using the equation below (Meadus et al., 2010), which is modified from the recommended method of AOAC.


$$\begin{array}{lllllll} IV= & \left(C16:1\times 0.95\right)+\left(C18:1\times 0.86\right)\; & +\left(C18:2\;\times 1.732\right)+\left(C18:3\times 2.616\right)\; & +\left(C20:1\times 0.795\right)+\left(C20:2\times 1.57\right)\;\; & +\left(C20:3\times 2.38\right)+\left(C20:4\times 3.19\right)\; & +\left(C20:5\times 4.01\right)+\left(C22:4\times 2.93\right)\; & +\left(C22:5\times 3.68\right)+(C22:6\times 4.64) \end{array}$$

### Analysis of Different Isoform of Vitamin E in Diets, Plasma, Liver and Loin Muscle

The concentration of different isoforms of tocopherol in plasma and liver samples was determined by the DSM research laboratory in Switzerland. Briefly, plasma or oil samples were mixed with milli-Q water and ethanol. Tocopherols were extracted from the aqueous suspension with hexane/BHT using a homogenizer. The tissue sample was saponified with potassium hydroxide solution in methanol. Tocopherols were then extracted from the saponified mixture with hexane/toluene. After centrifugation, an aliquot of the organic phase was injected onto a normal-phase HPLC system consisting of an auto-sampler, pump, and fluorescence detector. A Lichrosorb Si 60 normal-phase column (250 mm × 4 mm i.d.; particle size, 5 μm) was used. The mobile phase was a solution of 4.5% dioxane in hexane. The retention times for α-, β-, γ-, and δ-tocopherol were determined, respectively, with purified tocopherols of different isoforms with >95% purity from commercial sources. Quantification was performed by applying an external calibration.

### Antioxidant Capacity

Antioxidant measurements in the liver including superoxide dismutase (**SOD**) activity, MDA content, glutathione (**GSH**), and GSSG concentrations were analyzed with commercial assay kits purchased from Cayman (Ann Arbor, MI, USA). The SOD assay measurement measured three types of SOD (Cu/Zn, Mn, and Fe SOD).

### Swine Influenza Vaccination

As a means of evaluating potential impacts of the dietary treatments on the immune response, all pigs received two 2-mL doses of a commercial swine influenza vaccine (FluSure XP, Zoetis INC, Kalamazoo, MI, USA) administered intramuscularly on the right side of the neck; the two vaccinations were three weeks apart. This product contains four Influenza A Virus isolates including A/Swine/Iowa/110600/2000 (H1N1), A/Swine/Oklahoma/0726H/2008 (H1N2), A/Swine/North Carolina/394/2012 (H3N2), and A/Swine/ Minnesota/872/2012 (H3N2). Before injection, the freeze-dried vaccine was aseptically rehydrated with the accompanying adjuvant-containing sterile diluent. The first injection was on day 55 of the study, and the second injection was 3 wk afterward. Blood samples were collected by vena cava puncture immediately prior to each injection and 14 d after the second vaccination. After the blood collection, whole blood was transferred to 16 × 100 mm serum separator tubes (Becton, Dickinson and Company, Franklin Lakes, NJ) for serum separation. All blood samples were immediately placed on ice and then transported to the laboratory. Serum samples were obtained by centrifugation at 2,500 × *g* for 20 min at 4 °C; and then aliquoted into 1.5 mL Eppendorf Safe-Lock Tubes (Eppendorf North America, Hauppauge, NY), and stored at −80 °C until analysis.

### Serological Test

Serum samples were sent to the Veterinary Diagnostic Laboratory at the University of Minnesota for the serology test. They were firstly analyzed to screen the existence of antibodies to Swine influenza A virus with enzyme-linked immunosorbent assays (**ELISA**), the results were expressed as the ratio of ELISA optical densities for the specimen and the negative control (**S/N**) in a similar way used by Tse et al. (2012). Concentrations of antibodies to three popular viruses including IAV-S H3N2 Cluster IV, IAV-S Zoetis gamma H1 XP-012, and IAV-S Zoetis Delta 1 726H H1N2 were then measured using hemagglutination inhibition method, respectively.

### Statistical Analysis

Prior to analyses, all data were evaluated to identify any potential statistical outliers according to the test published by Barnett and Lewis (1994). Briefly, a set of data is ranked from low to high: *X*_L_, *X*_2_, …, *X*_H_, and the average and standard deviation are calculated, then suspected high or low outliers are tested by the following procedure: First, calculation of the statistic *T*: *T* = (*X*_H_ − Mean)/*s* for a high value, or *T* = (*X*_L_ − Mean)/*s* for the low value (where *s* refers to the standard deviation). Second, comparison of the value of *T* with the value from critical values for the 95% confidence interval was made (under conditions of this study, the critical value was 2.03). When the calculated *T* was larger than the critical value for the measurement, then the *X*_L_ or *X*_H_ was a potential outlier at the level of 5% significance. Potential outliers were then evaluated by reviewing study notes for that animal as well as response measures and laboratory values for other animals on that particular dietary treatment to determine if the value would be excluded from the data set.

Data analyses were performed in SAS (SAS Inst. Inc., Gary, NC) by least squares analysis of variance using the generalized linear model as a randomized complete block design. The individual pig served as the experimental unit. The model used was:


$$\begin{array}{lll} Y\;= & \;\mu +\;S_{l}+\;B_{i(l)}+\;V_{j}+\;F_{k}+\;VF_{jk}+\;SV_{lj}\; & +\;SF_{lk}+\;{\;\varepsilon \;}_{m(ijkl)}. \end{array}$$

In this equation, the parameters represent *Y* = response variables; µ = overall population mean; *S*_*l*_ = sex (male or female); *B*_*i*(*l*)_ = block (*i* = 1, 2, 3,…,0.6); *V*_*j*_ = VE level (11, 40, 100, or 200 ppm); *F*_*k*_ = fat sources (TW or CO).

When interactions between main effects were significant, further least squares mean separations were accomplished using the PDIFF option of SAS to analyze the treatment effects. For plasma α- and total-tocopherol data, there were interactions between time of blood collection and VE supplementation level for which a slope comparison with increasing time of supplementation of dietary VE was performed between VE supplementation levels by the CONTRAST option of SAS.

For immune response results, data transformation was applied to enable proper analysis, as all the antibody titer values were geometric means. Data were transformed with natural logarithm, the result with Negative (Neg) was applied the value of 2 to enable analytic comparison. *P* values from the ANOVA analysis of the transformed data were used to interpret potential treatment effects.

Statistically significant differences were established at *P* ≤ 0.05, tendencies were established at *P* ≤ 0.10 for main effects. Sex effects were observed but are not presented or discussed because when they were present they were similar to that which has previously been published elsewhere. To focus attention on statistically relevant data in the tables, any *P*-value greater than 0.10 was replaced with “—”.

## Results

### Analyzed Dietary VE Level and Fatty Acid Profile

The analyzed FA profile and VE content are listed in Table 1. The fat in the basal dietary ingredients, especially corn, plus the 5% added fat made the total dietary fat content around 7%. Thus, the dietary FA profiles were the result of both 5% added fat and ~2% oil in the basal diet. The differences in the FA profile were as expected, wherein the DCO treatments had more PUFA and less MUFA and SFA than the TW treatments. The ATA content in each diet was as designed and the other tocopherol contents were not different among treatments.

**Table 1.** Average fatty acid profile^1^ and vitamin E (VE) content of diets from phase 1 to phase 5 (as-fed basis)

Fat sources:	Tallow				Distiller’s corn oil			
VE (ATA), ppm:	11	40	100	200	11	40	100	200
Lipid, %	7.10	7.32	7.20	7.36	7.13	7.31	7.04	7.12
Fatty acid profile, %								
C14	2.07	1.96	2.04	1.98	0.06	0.05	0.05	0.05
C14:1	0.38	0.36	0.38	0.37	0.00	0.00	0.00	0.00
C16	21.19	20.65	21.10	20.72	12.31	13.15	13.20	13.22
C16:1	1.92	1.83	1.89	1.85	0.19	0.18	0.18	0.18
C17	0.87	0.83	0.86	0.84	0.09	0.08	0.08	0.08
C18	13.84	13.31	13.85	13.46	2.12	2.06	2.07	2.06
C18:1	35.09	34.69	35.16	34.84	26.23	25.92	25.87	26.03
C18:2n6	20.34	21.93	20.62	21.69	55.56	55.33	55.16	55.21
C18:3n3	1.09	1.02	1.09	1.08	2.15	2.13	2.17	2.10
C18:3n6	0.15	0.18	0.17	0.16	0.01	0.01	0.01	0.02
CLA	0.32	0.32	0.33	0.33	0.09	0.09	0.08	0.09
C20	0.36	0.36	0.23	0.26	0.55	0.41	0.49	0.40
C20:1	0.59	0.80	0.55	0.65	0.57	0.49	0.55	0.50
C20:3	0.06	0.06	0.06	0.15	0.02	0.03	0.01	0.01
∑SFA	38.67	37.44	38.40	37.57	15.12	15.76	15.90	15.81
∑MUFA	38.46	38.14	38.45	38.17	27.03	26.63	26.64	26.75
∑PUFA	21.97	23.51	22.26	23.42	57.84	57.59	57.44	57.43
IV	72.50	74.84	73.02	74.82	125.34	124.54	124.31	124.33
Tocopherol isoforms, ppm								
ATA	12.60	33.00	87.80	171.00	14.40	42.40	96.60	192.60
Alpha	5.00	6.40	5.00	4.40	11.20	10.00	11.00	11.00
Beta	1.25	2.00	1.20	1.20	4.50	4.25	4.75	4.50
Gamma	25.80	31.80	24.20	23.60	38.20	34.00	36.20	36.00
Delta	7.40	8.80	6.80	6.20	4.80	6.20	6.20	6.40

^1^Values are average of diets from five phases. ATA, α-tocopheryl-acetate.

### Intake of Different Fatty Acids

The analyzed dietary FA profiles for each growth phase for each fat source were averaged (because all four vitamin E treatments were developed from a single basal fat source mixing), then were multiplied by the daily feed intake and summed to yield the total fatty acid intake during the period of growth on the study for each individual pig. As shown in Table 2, the intake of total lipid did not differ, but the intake of all individual FAs differed (*P* < 0.01) between pigs fed TW and DCO diets. Pigs fed the TW diet had higher intake of total SFA (*P* < 0.01) and total MUFA (*P* < 0.01) but lower intake of total PUFA (*P* < 0.01) than the pigs fed the DCO diet. For the pigs fed the TW diet, the intake of all SFA including C14 (*P* < 0.01), C16 (*P* < 0.01), C17 (*P* < 0.01), and C18 (*P* < 0.01) were higher except for C20 (*P* < 0.01), which was lower than that of pigs fed DCO diets. The intake of all the individual MUFA including C14:1, C16:1, C17:1, C18:1, C20:1 were higher in pigs fed TW diets than that of pigs fed DCO diets. For the intake of PUFA, the intake of C18:2 and C18:3n-3 was lower while C18:3n-6 and C20:3 were higher in pigs fed TW diets than that of pigs fed DCO diets. The intake of all FAs was not affected by levels of dietary VE.

**Table 2.** Effect of different fat sources and vitamin E (VE) supplementation levels on intake of individual fatty acids^1^ in pigs from 28 to 150 kg

Fat source:	TW				DCO				SEM	*P*-value		
1VE (ATA), ppm:	11	40	100	200	11	40	100	200		VE^2^		Fat
										L	Q	
Lipid, kg	22.33	22.16	23.16	21.60	23.48	22.65	22.65	22.36	0.60	—	—	—
Individual fatty acid, g												
C14	450.65	447.22	467.27	435.77	12.30	11.86	11.86	11.71	8.73			
C14:1	83.10	82.47	86.17	80.36	0.00	0.00	0.00	0.00	1.61	—	—	<0.01^3^
C16	4,676.26	4,640.63	4,848.74	4,521.85	3,065.83	2,956.47	2,957.13	2,918.89	107.70	—	—	<0.01^3^
C16:1	419.26	416.07	434.72	405.42	42.69	41.17	41.18	40.64	8.20	—	—	<0.01^3^
C17	191.36	189.90	198.42	185.04	19.17	18.48	18.49	18.25	3.74	—	—	<0.01^3^
C18	3,041.44	3,018.27	3,153.63	2,941.02	485.73	468.41	468.51	462.45	60.06	—	—	<0.01^3^
C18:1	7,798.65	7,739.23	8,086.30	7,541.14	6,114.99	5,896.86	5,898.18	5,821.91	189.27	—	—	<0.01^3^
C18:2n-6	4,729.27	4,693.24	4,903.71	4,573.11	12,975.87	12,513.01	12,515.81	12,353.96	241.86	—	—	<0.01^3^
C18:3n-3	241.54	239.70	250.45	233.56	498.33	480.56	480.67	474.45	9.87	—	—	<0.01
C18:3n-6	37.21	36.93	38.58	35.98	3.27	3.15	3.15	3.11	0.73	—	—	<0.01
CLA	71.64	71.10	74.29	69.28	20.72	19.98	19.99	19.73	1.46	—	—	<0.01^3^
C20	65.16	64.66	67.56	63.01	103.59	99.89	99.92	98.62	2.22	—	—	<0.01^3^
C20:1	142.58	141.49	147.84	137.87	122.01	117.66	117.68	116.16	3.57	—	—	<0.01
C20:3	16.09	15.97	16.68	15.56	4.40	4.24	4.25	4.19	0.33	—	—	<0.01^3^
∑SFA	8,496.37	8,431.63	8,809.75	8,215.82	3,687.58	3,556.04	3,556.83	3,510.84	180.40	—	—	<0.01^3^
∑MUFA	8,548.01	8,482.88	8,863.30	8,265.76	6,288.82	6,064.49	6,065.84	5,987.40	203.34	—	—	<0.01^3^
∑PUFA	5,095.75	5,056.93	5,283.71	4,927.50	13,502.60	13,020.94	13,023.86	12,855.44	253.24	—	—	<0.01^3^

^1^Values are average of six replicates. ATA, α-tocopheryl-acetate; TW, tallow; DCO, distiller’s corn oil. *P*-values greater than 0.10 were replaced with “—”.

^2^Linear (L) and quadratic (Q) responses based on dietary VE levels.

^3^Interaction between fat and dietary VE level, *P* < 0.05.

### Fatty Acid Profile in the Backfat

Table 3 shows the effects of dietary VE (i.e., ATA) supplementation and fat sources on the FA profile of the backfat. No interactions between dietary VE levels and fat sources were observed on FA profile in the backfat at slaughter. Significant differences (*P* < 0.05) were detected among pigs fed dietary fat sources on all the FAs analyzed except for C10. Corresponding to the difference in the intake of different FAs, pigs fed the TW diet had more SFA (*P* < 0.01) including C12 (*P* < 0.05), C14 (*P* < 0.05), C16 (*P* < 0.01), C17 (*P* < 0.01), and C18 (*P* < 0.01) than the pigs fed the DCO diet except for C20 (*P* < 0.05), which was lower than the pigs fed the DCO diet in the backfat. Pigs fed the TW diet also had more MUFA (*P* < 0.01) including C14:1 (*P* < 0.01), C16:1 (*P* < 0.01), C17:1 (*P* < 0.01), C18:1 (*P* < 0.01), and C20:1 (*P* < 0.01) than the pigs fed the DCO diet in the backfat. The concentrations of PUFA including C18:2n-6, C18:3n-3, C20:2, C20:3, and C20:4 in the backfat were greater (*P* < 0.01) in the pigs fed the DCO diet than that of the pigs fed the TW diet except for C18:3n-6 and CLA, which was lower than the pigs fed the TW diet.

**Table 3.** Effect of different fat sources and vitamin E (VE) supplementation levels on fatty acid profile^1^ of the backfat

Fat source:	TW				DCO				SEM	*P*-value		
VE (ATA), ppm:	11	40	100	200	11	40	100	200		VE^2^		Fat
										L	Q	
C10	0.05	0.05	0.04	0.05	0.05	0.05	0.05	0.05	0.003	—	—	—
C12	0.06	0.06	0.06	0.06	0.06	0.06	0.06	0.05	0.003	—	—	0.02
C14	1.41	1.39	1.36	1.37	1.04	1.03	1.09	1.00	0.047	—	—	<0.01
C14:1	0.05	0.05	0.05	0.05	0.01	0.01	0.01	0.00	0.003	—	—	<0.01
C16	22.98	22.94	22.81	22.59	20.42	20.19	20.94	20.29	0.407	—	—	<0.01
C16:1	2.62	2.57	2.37	2.61	1.58	1.59	1.55	1.48	0.100	—	—	<0.01
C17	0.56	0.51	0.53	0.51	0.18	0.20	0.19	0.20	0.023	—	—	<0.01
C18	13.24	12.99	14.14	13.23	10.73	10.87	11.48	11.77	0.442	—	—	<0.01
C18:1	45.72	46.58	45.63	46.08	37.32	37.31	37.27	37.38	0.518	—	—	<0.01
C18:2n-6	9.01	8.54	8.62	9.05	24.54	24.81	23.54	23.91	0.676	—	—	<0.01
C18:3n-3	0.39	0.37	0.38	0.38	0.77	0.79	0.68	0.74	0.030	—	—	<0.01
C18:3n-6	0.19	0.19	0.19	0.20	0.07	0.07	0.06	0.07	0.008	—	—	<0.01
CLA	0.40	0.39	0.38	0.38	0.06	0.06	0.12	0.07	0.027	—	—	<0.01
C20	0.21	0.23	0.24	0.25	0.26	0.24	0.28	0.26	0.016	0.06	—	0.02
C20:1	0.95	1.04	1.07	1.08	0.89	0.82	0.89	0.82	0.055	—	—	<0.01
C20:2	0.32	0.33	0.33	0.35	1.16	1.12	1.10	1.12	0.022	—	—	<0.01
C20:3	0.068	0.057	0.058	0.051	0.098	0.082	0.089	0.092	0.006	—	—	<0.01
C20:4	0.14	0.13	0.14	0.13	0.22	0.23	0.21	0.23	0.009	—	—	<0.01
∑SFA	38.57	38.24	39.26	38.15	32.77	32.67	34.12	33.64	0.554	—	0.10	<0.01
∑MUFA	49.85	50.73	49.60	50.30	39.94	39.89	39.87	39.83	0.579	—	—	<0.01
∑PUFA	10.05	9.62	9.73	10.16	26.82	27.09	25.61	26.16	0.712	—	—	<0.01
IV	61.18	61.17	60.37	61.73	81.81	82.28	79.58	80.57	1.000	—	—	<0.01

^1^Values are average of six replicates. ATA, α-tocopheryl-acetate; TW, tallow; DCO, distiller’s corn oil. *P*-values greater than 0.10 were replaced with “—”.

^2^Linear (L) and quadratic (Q) responses based on dietary VE levels. No interactions between fat sources and dietary VE levels were observed.

No effect of dietary VE supplementation levels was observed on FA profile in the backfat except that increasing dietary VE supplementation levels increased C20:0 (linear, *P* = 0.06) and SFA (quadratic, *P* = 0.10).

### Fatty Acid Profile in the Belly Fat

In Table 4, the FA profile of belly fat was similarly affected by dietary treatment as occurred in the backfat. No interactions between dietary VE supplementation levels and fat sources were observed on FA profile in the belly fat at slaughter. As expected, differences (*P* < 0.05) were detected among pigs fed dietary fat sources on most FAs analyzed; pigs fed the TW diet had more SFA (*P* < 0.01) including C12 (*P* = 0.07), C14 (*P* < 0.01), C16 (*P* < 0.01) and C18 (*P* < 0.01) than pigs fed the DCO diet except for C20:0, which was lower than the pigs fed the DCO diet (*P* = 0.10). Corresponding to the difference in the intake of different FAs, the concentrations of MUFA including C14:1, C16:1, C18:1, and C20:1 were greater in pigs fed the TW diet compared to pigs fed the DCO diet. Corresponding to the intake of individual FAs, the concentrations of PUFAs including C18:2n-6 (*P* < 0.01), C18:3n-3 (*P* < 0.01), C18:3n-6 (*P* < 0.01), C20:2 (*P* < 0.01), C20:3 (*P* < 0.01), and C20:4 (*P* < 0.01) were greater in the belly fat of pigs fed the DCO diet compared to that of pigs fed the TW diet except for CLA that was lower in the pigs fed the DCO diet compared to those fed the TW diet (*P* < 0.01).

**Table 4.** Effect of different fat sources and vitamin E (VE) supplementation levels on fatty acid profile^1^ of the belly fat

Fat source:	TW				DCO				SEM	*P*-value		
VE (ATA), ppm:	11	40	100	200	11	40	100	200		VE^2^		Fat
										L	Q	
C10	0.06	0.06	0.05	0.06	0.05	0.06	0.06	0.06	0.003	—	—	—
C12	0.07	0.07	0.07	0.07	0.06	0.07	0.07	0.06	0.003	—	—	0.07
C14	1.50	1.49	1.44	1.45	1.18	1.19	1.25	1.15	0.045	—	—	<0.01
C14:1	0.05	0.05	0.04	0.05	0.01	0.01	0.01	0.01	0.003	—	—	<0.01
C16	22.87	22.14	22.64	22.74	21.04	20.68	20.91	20.45	0.423	—	—	<0.01
C16:1	3.09	3.17	2.77	2.87	2.02	2.05	2.09	1.87	0.113	0.04	—	<0.01
C17	0.44	0.38	0.39	0.41	0.15	0.16	0.15	0.18	0.026	—	—	<0.01
C18	11.29	10.24	11.57	11.82	9.70	9.53	9.52	10.07	0.427	0.08	—	<0.01
C18:1	48.69	50.74	49.62	48.64	41.55	41.51	42.58	40.86	0.899	—	—	<0.01
C18:2n-6	7.89	7.60	7.41	7.91	20.41	21.01	19.53	21.47	0.661	—	—	<0.01
C18:3n-3	0.35	0.33	0.34	0.32	0.62	0.69	0.63	0.69	0.033	—	—	<0.01
C18:3n-6	0.01	0.01	0.01	0.04	0.03	0.04	0.03	0.05	0.009	0.03	—	<0.01
CLA	0.40	0.41	0.36	0.37	0.07	0.07	0.07	0.07	0.011	0.08	0.10	<0.01
C20	0.20	0.20	0.21	0.21	0.22	0.21	0.24	0.21	0.015	—	—	0.10
C20:1	1.02	1.09	1.15	1.06	0.98	0.87	1.00	0.85	0.074	—	—	<0.01
C20:2	0.31	0.32	0.32	0.31	1.04	1.01	1.04	1.06	0.038	—	—	<0.01
C20:3	0.07	0.06	0.06	0.06	0.10	0.09	0.09	0.10	0.008	—	—	<0.01
C20:4	0.15	0.15	0.15	0.13	0.24	0.25	0.22	0.26	0.011	—	—	<0.01
∑SFA	36.50	34.65	36.43	36.83	32.43	31.92	32.22	32.21	0.727	—	—	<0.01
∑MUFA	53.34	55.53	54.03	53.07	44.70	44.60	45.85	43.76	0.973	—	—	<0.01
∑PUFA	9.18	8.89	8.55	9.14	22.52	23.15	21.62	23.70	0.701	—	—	<0.01
IV	62.55	63.91	61.96	62.18	78.39	79.48	77.81	79.73	0.907	—	—	<0.01

^1^Values are average of 6 replicates. ATA, α-tocopheryl-acetate; TW, tallow; DCO, distiller’s corn oil. *P*-values greater than 0.10 were replaced with “—”.

^2^Linear (L) and quadratic (Q) responses based on dietary VE levels. No interactions between fat sources and dietary VE levels were observed.

Dietary VE supplementation levels only affected the concentration of C16:1 (linear, *P* < 0.05), C18:0 (linear, *P* = 0.08), C18:3n-6 (linear, *P* < 0.05), and CLA (linear and quadratic, *P* < 0.10) in the belly fat, wherein the concentration of C16:1 and CLA decreased, and the concentration of C18:0 and C18:3n-6 increased with the increasing dietary VE supplementation levels from 11 to 200 ppm.

### Fatty Acid Profile in the Liver

As shown in Table 5, no interactions between dietary VE supplementation levels and fat sources were observed on FA profile in the liver at slaughter except C16:1 (*P* < 0.05). Generally corresponding to the intake of FA, differences (*P* < 0.05) were detected among pigs fed dietary fat sources on all FA except for C20:1, CLA, and C20:4.

**Table 5.** Effect of different fat sources and vitamin E (VE) supplementation levels on fatty acid profile^1^ of the liver

Fat source:	TW				DCO				SEM	*P*-value		
VE (ATA), ppm:	11	40	100	200	11	40	100	200		VE^2^		Fat
										L	Q	
Lipid, %	1.41	1.63	1.55	1.39	1.47	1.49	1.34	1.79	0.117	—	—	—
Fatty acid profile, %												
C14	0.51	0.49	0.60	0.49	0.40	0.50	0.36	0.36	0.049	—	—	<0.01
C16	14.68	13.94	14.92	14.70	13.11	13.84	13.32	12.92	0.392	—	—	<0.01
C16:1	1.48	1.35	1.57	1.26	0.43	0.68	0.35	0.49	0.103	—	—	<0.01^3^
C17	0.69	0.71	0.69	0.66	0.54	0.56	0.56	0.60	0.060	—	—	<0.01
C18	19.25	19.22	19.05	18.85	19.33	19.72	20.50	19.93	0.497	—	—	0.04
C18:1	16.96	15.57	17.00	16.39	11.09	11.89	11.07	11.37	0.562	—	—	<0.01
C18:2n-6	15.25	14.81	14.75	15.44	23.49	23.51	23.50	22.39	0.483	—	—	<0.01
C18:3n-3	0.24	0.23	0.23	0.23	0.39	0.35	0.32	0.32	0.025	—	—	<0.01
C18:3n-6	0.18	0.19	0.20	0.19	0.25	0.26	0.22	0.22	0.031	—	—	0.02
CLA	1.20	1.24	1.26	1.32	1.15	1.05	1.22	1.15	0.185	—	—	—
C20:1	0.27	0.16	0.18	0.20	0.15	0.21	0.19	0.14	0.048	—	—	—
C20:2	0.40	0.33	0.39	0.42	0.77	0.64	0.71	0.71	0.058	—	—	<0.01
C20:3	1.21	0.85	0.79	0.75	0.76	0.55	0.85	0.76	0.106	—	—	0.03
C20:4	18.22	20.39	18.97	19.02	19.22	19.24	19.44	19.57	0.677	—	—	—
C22	0.38	0.32	0.27	0.24	0.14	0.11	0.20	0.13	0.033	0.07	-	<0.01
C22:2	2.16	2.06	2.27	2.18	1.74	1.77	1.73	2.10	0.119	—	—	<0.01
C22:4	0.87	0.76	0.82	0.78	0.95	0.94	0.98	1.09	0.084	—	—	<0.01
C22:5	1.41	1.42	1.38	1.49	1.11	1.07	1.06	1.31	0.092	0.10	—	<0.01
C22:6	0.80	1.09	0.89	0.83	0.71	0.44	0.57	0.63	0.141	—	—	<0.01
∑SFA	35.45	34.69	35.54	34.94	33.51	34.72	34.87	33.95	0.629	—	—	0.02
∑MUFA	18.72	17.08	18.75	17.85	11.67	12.79	11.61	12.00	0.655	—	—	<0.01
∑PUFA	41.93	43.37	41.94	42.22	50.92	49.82	50.61	50.25	0.807	—	—	<0.01
IV	122.06	126.85	123.06	122.58	132.66	130.22	131.39	131.94	1.804	—	—	<0.01

^1^Values are average of 6 replicates. ATA, α-tocopheryl-acetate; TW, tallow; DCO, distiller’s corn oil. *P*-values greater than 0.10 were replaced with “—”.

^2^Linear (L) and quadratic (Q) responses based on dietary VE levels.

^3^Interaction between fat and dietary VE level, *P* < 0.05.

Regarding the fat source effect, the pigs fed the TW diet had greater content of SFA (*P* < 0.05) and MUFA (*P* < 0.01), while lower PUFA (*P* < 0.01) and IV (*P* < 0.01) in the liver than the pigs fed the DCO diet. Concentrations of most SFA including C14 (*P* < 0.01), C16 (*P* < 0.01), C17 (*P* < 0.01), and C22 (*P* < 0.01) except for C18, and MUFA including C16:1 (*P* < 0.01) and C18:1 (*P* < 0.01) were greater in the pigs fed the TW diet compared to the pigs fed the DCO diet. Concentrations of PUFA including C18:2n-6 (*P* < 0.01), C18:3n-3 (*P* < 0.01), C18:3n-6 (*P* < 0.05), C20:2 (*P* < 0.01), and C22:4 (*P* < 0.01) were greater in the pigs fed the DCO diet than the pigs fed the TW diet. However, the concentrations of C20:3 (*P* < 0.05), C22:2 (*P* < 0.01), C22:5 (*P* < 0.01), and C22:6 (*P* < 0.01) were greater in the pig fed the TW diet than the DCO diet whereas C18:0 (*P* < 0.05) was greater in the pigs fed the DCO diet than the TW diet.

Dietary VE supplementation levels did not affect FA profile in the liver except for C22:0 and C22:5 (linear, *P* < 0.10) in which the concentration of C22:0 decreased, and the concentration of C22:5 increased with the increasing dietary VE supplementation levels from 11 to 200 ppm.

### Plasma VE Concentrations

Table 6 shows the effect of different fat sources and dietary VE supplementation levels on VE concentration in plasma. No interactions between fat sources and dietary VE supplementation levels were observed on plasma α-tocopherol (**α-T**) and total tocopherol concentrations. Increasing dietary VE supplementation levels from 11 to 200 ppm increased α-T concentration in plasma at the end of phase 1 (linear, *P* < 0.01) and phases 2, 3, 4, and 5 (linear and quadratic, *P* < 0.01). The α-T concentration in plasma was greater in pigs fed the TW diet than DCO diet in phase 3 (*P* < 0.05). Because α-T concentrations were the primary isoform detected, total-tocopherol concentrations showed the same patterns as the α-T concentrations. With the increasing time of supplementation of dietary VE, the concentration of plasma α-T increased (linear and quadratic, *P* < 0.01) with significant interaction with levels of VE. The interaction was primarily a result of the pigs fed 11 ppm dietary VE supplementation level had a smaller (*P* < 0.05) slope than pigs from the other VE treatments. The trend of plasma α-T concentration along with time was not affected by fat sources.

**Table 6.** Effect of different fat sources and vitamin E (VE) supplementation levels on VE concentration^1^ in plasma

Fat source:	TW				DCO				SEM	*P*-value		
VE (ATA), ppm:	11	40	100	200	11	40	100	200		VE^2^		Fat
										L	Q	
α-Tocopherol^3^, µg/mL												
Day 0	1.55	1.39	1.45	1.40	1.67	1.47	1.22	1.96	0.243	—	—	—
Phase 1	1.55	2.28	2.83	3.50	1.85	2.66	2.75	4.02	0.269	<0.01	—	—
Phase 2	1.60	2.60	3.55	3.61	1.82	2.75	3.08	3.80	0.244	<0.01	<0.01	—
Phase 3	2.23	3.64	3.86	4.31	2.22	3.07	3.43	3.66	0.267	<0.01	<0.01	0.03
Phase 4	2.01	3.31	4.13	4.47	2.33	3.06	3.59	4.17	0.214	<0.01	<0.01	—
Phase 5	2.60	3.92	4.41	4.36	2.54	3.30	4.25	4.82	0.362	<0.01	<0.01	—
γ-Tocopherol^4^^,^^5^, µg/mL												
Day 0	nd^6^	nd	nd	nd	nd	nd	nd	nd	—	—	—	—
Phase 1	0.35	0.17	0.03	0.05	0.22	0.27	nd	nd	0.051	<0.01	<0.01	—
Phase 2	0.24	0.23	0.05	0.04	0.25	0.19	0.09	0.08	0.046	<0.01	0.04	—
Phase 3	0.30	0.28	0.17	0.04	0.24	0.15	0.17	0.10	0.033	<0.01	—	—^I^
Phase 4	0.35	0.22	0.03	nd	0.33	0.24	0.10	0.03	0.077	<0.01	0.07	—
Phase 5	0.35	0.23	nd	nd	0.12	0.07	0.12	nd	0.076	<0.01	—	—
Total-tocopherol^6^, µg/mL												
Day 0	1.55	1.39	1.45	1.40	1.67	1.47	1.22	1.96	0.244	—	—	—
Phase 1	1.90	2.45	2.86	3.56	2.07	2.93	2.75	4.02	0.271	<0.01	—	—
Phase 2	1.84	2.82	3.60	3.65	2.07	2.94	3.17	3.89	0.258	<0.01	<0.01	—
Phase 3	2.52	3.92	4.03	4.35	2.46	3.22	3.61	3.76	0.280	<0.01	<0.01	0.03
Phase 4	2.36	3.53	4.16	4.47	2.66	3.30	3.69	4.21	0.242	<0.01	<0.01	—
Phase 5	2.95	4.15	4.41	4.36	2.66	3.37	4.37	4.82	0.391	<0.01	0.02	—

^1^Values are the average of six replicates; β-tocopherol and δ-tocopherol were not detected in any sample from day 0 to the end of each phase. ATA, α-tocopheryl-acetate; TW, tallow; DCO, distiller’s corn oil. *P*-value greater than 0.10 was replaced with “—”.

^2^Linear (L) and quadratic (Q) responses based on dietary VE levels. ^I^ Interaction between fat and dietary VE level, *P* < 0.05.

^3^Time effect, linear and quadratic, *P* < 0.01; interaction between time and dietary VE levels, *P* < 0.01. No interaction between time and fat sources was observed.

^4^Time effect, linear and quadratic, *P* < 0.01; interaction between time and dietary VE levels, *P* < 0.05. No interaction between time and fat sources was observed.

^5‘^nd’ means not detectable, for analysis purpose, a value of 0.001 was applied for those ones with ‘nd’.

^6^Time effect, linear and quadratic, *P* < 0.01; interaction between time and dietary VE levels, *P* < 0.01. No interaction between time and fat sources was observed.

### Vitamin E Concentration in Liver, Loin Muscle, and Antioxidant Capacity

The concentrations of VE in liver and loin muscle, and liver antioxidant capacity are presented in Table 7. No β-tocopherol or δ-tocopherol was detected in liver and loin muscle. Increasing dietary VE supplementation levels from 11 to 200 ppm increased α-T and total-tocopherol concentrations (linear, *P* < 0.05) in liver and loin muscle, and decreased γ-T concentration in the liver (linear, *P* = 0.06) and loin muscle (linear and quadratic, *P* < 0.05). Interactions between fat sources and dietary VE supplementation levels were observed in the concentration of γ-T in the liver (*P* < 0.05) and α-T in the loin muscle (*P* < 0.05), wherein the pigs fed the TW diet had greater γ-T concentration in the liver (*P* < 0.05) than the pigs fed the DCO diet when the lower levels of VE (11 and 40 ppm) was supplemented and increasing dietary VE supplementation level increased α-T concentration in the loin muscle of the pigs fed the TW diet with a greater degree compared to the pigs fed the DCO diet. The pigs fed the TW diet had greater α-T (*P* < 0.05), γ-T (loin muscle only, *P* = 0.09), and total (*P* < 0.05) tocopherol concentrations than the pigs fed the DCO diet.

**Table 7.** Effect of different fat sources and vitamin E (VE) supplementation levels on VE concentration^1^ in the liver and loin muscle and antioxidant capacity

Fat source:	TW				DCO				SEM	*P*-value		
VE (ATA), ppm:	11	40	100	200	11	40	100	200		VE^2^		Fat
										L	Q	
Liver, µg/g wet liver												
α-Tocopherol	5.82	13.14	17.52	30.74	6.98	10.64	15.04	22.45	2.055	<0.01	—	0.04
γ-Tocopherol	0.82	0.83	0.20	0.57	0.66	0.59	0.78	0.29	0.157	0.06	—	—^3^
Total-tocopherol	6.64	13.97	17.72	31.31	7.64	11.23	15.82	22.73	2.090	<0.01	—	0.05
Loin muscle, µg/g wet tissue												
α-Tocopherol	4.07	5.59	6.72	8.71	4.41	4.90	5.31	6.29	0.465	<0.01	—	<0.01^3^
γ-Tocopherol	1.21	0.91	0.26	0.19	0.97	0.44	0.16	0.13	0.181	<0.01	0.01	0.09
Total-tocopherol	5.28	6.50	7.43	9.47	5.39	5.35	5.47	7.01	0.497	<0.01	—	<0.01
Liver antioxidant capacity												
GSH µmol/g protein	88.15	99.38	90.88	85.05	100.20	85.80	97.76	91.99	8.319	—	—	—
GSSG µmol/g protein	8.08	8.47	7.20	7.00	9.44	6.94	8.39	7.57	1.038	0.07	—	—
GSH/GSSG	12.06	12.22	15.19	12.58	11.45	15.37	12.36	12.26	1.024	—	—	—
SOD, U/mg protein	35.98	41.96	42.92	37.20	37.36	36.26	41.30	41.65	2.093	0.10	0.03	—
MDA, nmol/g protein	90.11	74.04	79.05	76.12	88.42	68.69	69.58	79.03	6.972	0.04	0.02	—

^1^Values are the average of 6 replicates; β-tocopherol and δ-tocopherol were not detected in liver or muscle. ATA, α-tocopheryl-acetate; TW, tallow; DCO, distiller’s corn oil. *P*-values greater than 0.10 were replaced with “—”.

^2^Linear (L) and quadratic (Q) responses based on dietary VE levels.

^3^Interaction between fat and dietary VE level, *P* < 0.05.

Regarding antioxidant activity in liver, no effect of dietary fat sources and interaction between fat sources and dietary VE supplementation levels were detected on liver antioxidant capacity when measuring the content of GSH, GSSG, and MDA, and activity of SOD. Increasing dietary VE supplementation levels from 11 to 200 ppm increased SOD activity (quadratic, *P* < 0.05; highest at 100 ppm), and decreased MDA content (quadratic, *P* < 0.05; lowest at 40 ppm) and GSSG content (linear, *P* = 0.07) in the liver.

### Immune Response

As shown in Table 8, the FluSure vaccination was proven to be effective in generating particular antibodies against the virus included in the vaccine as supported by the large increase in antibody content following the second vaccination. No effect of dietary fat sources, VE supplementation levels, and no interactions between dietary VE treatments and fat sources were detected on the immune response when measured by the content of antibody titers with the exception that the antibody titer measured by ELISA was greater in the pigs fed the DCO diet than those fed the TW diet at day 0 (*P* < 0.05) and that linear increases were observed in antibody titer measured for H1N2 (*P* < 0.05) and H1 (*P* = 0.09) with increasing VE supplementation levels at day 21. An interaction (*P* < 0.05) was observed in antibody titer measured by ELISA at day 36 wherein the antibody titer decreased with increasing dietary VE supplementation level only within the TW group.

**Table 8.** Effect of different fat sources and vitamin E (VE) supplementation levels on antibody titers^1^ of pigs to swine influenza^2^ vaccination

Fat source:	TW				DCO				SEM	*P*-value		
VE (ATA), ppm:	11	40	100	200	11	40	100	200		VE^3^		Fat
										L	Q	
Day 0												
ELISA	0.83	0.77	0.85	0.79	0.91	0.93	0.77	0.88	0.035	—	—	0.03
H1N2	Neg	Neg	Neg	Neg	Neg	Neg	Neg	Neg	—	—	—	—
H1	Neg	Neg	Neg	Neg	Neg	Neg	Neg	Neg	—	—	—	—
H3N2	Neg	Neg	Neg	Neg	Neg	Neg	Neg	Neg	—	—	—	—
Day 21												
ELISA	0.80	0.71	0.78	0.78	0.86	0.75	0.73	0.77	0.050	—	—	—
H1N2	5.85	6.03	5.02	2.62	5.02	6.56	2.76	2.76	2.683	0.01	—	—
H1	4.31	5.02	2.62	Neg	5.63	7.65	Neg	6.03	3.317	0.09	—	—
H3N2	Neg	Neg	Neg	Neg	2.62	Neg	Neg	Neg	0.615	—	—	—
Day 36												
ELISA	0.32	0.17	0.22	0.25	0.23	0.22	0.27	0.22	0.057	—	—	-^4^
H1N2	160.00	139.29	113.14	113.14	80.00	113.14	121.26	121.26	50.36	—	—	—
H1	126.99	183.79	142.54	179.59	113.14	142.54	105.56	160.00	29.60	—	—	—
H3N2	80.00	121.26	126.99	80.00	44.90	80.00	80.00	105.56	32.86	—	—	—

^1^Values for ELISA are the average of 6 replicates, values for all the antibody titer values were geometric means, data were transformed with natural logarithm for analysis. Result with Negative (Neg) was applied the value of 2 to enable ANOVA analysis. VE, vitamin E; ATA, α-tocopheryl-acetate; TW, tallow; DCO, distiller’s corn oil. *P*-value greater than 0.10 was replaced with “—”.

^2^ELISA results were expressed as the ratio of ELISA optical densities for the specimen and the negative control (S/N). Titer of three virus antibodies to IAV-S Zoetis Delta 1 726H H1N2 (H1N2), IAV-S Zoetis gamma H1 XP-012 (H1), and IAV-S H3N2 Cluster IV (H3N2) was analyzed, respectively.

^3^Linear (L) and quadratic (Q) responses based on dietary VE levels.

^4^Interaction between fat and dietary VE level, *P* < 0.05.

## Discussion

In the current research, largely corresponding to the intake of different individual FAs, the pigs fed the TW diet had greater SFA including C12:0, C14:0, C16:0, and C18:0, and MUFA including C14:1, C16:1, C18:1, and C20:1, lower PUFA including C18:2n-6, C18:3n-3, C20:2, C20:3 and C20:4 and IV in backfat and belly fat compared to the pigs fed the DCO diet. As reported in our previous study (Wang et al., 2022b), it has been documented that changes in the FA composition of swine diets can alter the FA profile in adipose tissues of pigs as portions of non-essential FAs are incorporated into lipid tissues of pigs as has been previously demonstrated (Rentfrow et al., 2003; Duran-Montgé et al., 2009). However, Duran-Montgé et al. (2009) reported that changes in dietary PUFA content can alter tissue FA composition to a greater extent compared to SFA and MUFA and, also, that the dietary fat source and content affected the FA composition even at a similar level of total FA intake. The current study agrees with the findings in Duran-Montgé et al. (2009). The main differences in FA composition between the two fat sources used in the current study, TW and DCO, as stated were greater content of SFA and MUFA but lower content of PUFA in TW than DCO.

Linoleic acid (C18:2n-6) is the most abundant FA in the DCO diets, which resulted in ~2.6 times greater intake in the DCO treatment than the TW treatment. A similar proportional difference was observed in the linoleic acid concentrations in backfat and belly fat indicating concentration-dependent deposition of linoleic acid in the adipose tissues that agree with Wang et al. (2022b). Additionally, the concentration of the other PUFA including C18:3n-6, C18:3n-3, and CLA reflected the intake of those individual FAs. The extent of the change in PUFA content in adipose tissues such as linoleic acid was always the greatest among all FA (Duran-Montgé et al., 2009).

As a MUFA, oleic acid (C18:1) is the most abundant FA found in the adipose tissues of pigs. In the current study, the changes in oleic acid composition between TW and DCO groups were not as great as the linoleic acid composition change. While the intake of oleic acid with the TW diet was ~1.3 times greater than the DCO diet, the increase in the concentration of oleic acid in backfat and belly fat in the pigs fed the TW diet was somewhat less but similar, being ~1.2 times greater than the pigs fed the DCO diet, again similar with Wang et al. (2022b). It could be explained by potentially reduced de novo synthesis of oleic acid from SFA in adipose tissues of pigs fed the TW diet due to high MUFA intake that could reduce stearoyl-CoA desaturase gene expression that is responsible for catalyzing the conversion of SFA to MUFA (Kellner et al., 2017).

The other two main FA found in the adipose tissues of pigs are the SFAs palmitic acid (C16:0) and stearic acid (C18:0). The composition of palmitic and stearic acids changed consistently with directional changes in these FAs in the diets but to a much smaller extent than oleic acid and linoleic acid. In this study, the intake of palmitic and stearic acids were 160% and 650% times greater in the pigs fed the TW diet than the DCO diet, respectively, while only 8% to 12% and 15% to 20% increases were detected in the concentration of palmitic and stearic acids in the adipose tissues (backfat and belly fat) between the TW and DCO treatments, respectively. This result regarding palmitic acid agrees with previous studies in which only small differences (2%), although significant, were observed in palmitic acid content when supplementation of animal fats (beef tallow, poultry fat, and choice white grease, 5%) was compared to soybean oil (5%) and no fat-added diets (Apple et al., 2009a; b; Benz et al., 2011), and when 5% linseed oil supplementation was compared to 5% olive oil supplementation (Nuernberg et al., 2005). A lower stearic acid content in adipose tissue of pigs has also been reported when animal fats were supplemented to the diets compared to vegetable oils such as sunflower oil, soybean oil, and corn oil (Mitchaothai et al., 2007; Apple et al., 2009a, 2009b). As palmitic and stearic acids can be actively synthesized endogenously in the body (Świątkiewicz et al., 2021), even though their composition did change consistently herein when dietary fat sources changed, the modification of these FA by dietary treatment is somewhat limited. Therefore, these results indicate that the SFA composition in the adipose tissues of pigs is less susceptible to change compared with MUFA and PUFA composition even when the diets contained different levels of SFA as existed between TW and DCO diets used in the current study. Thus, changes of major SFA, MUFA, and PUFA in adipose tissues of pigs are affected both by changes in dietary FA intake together with the active synthesis and metabolism of FA in the adipose tissues (Ramsay et al., 2001; Kloareg et al., 2007).

Regarding IV, the higher IV value of backfat and belly fat was observed when pigs were fed the DCO diet compared to the pigs fed the TW diet, which reflects the greater PUFA content in the DCO group than the TW group. This result agrees with our previous study (Wang et al., 2022b) and demonstrated, again, the collective effect of the change in dietary PUFA content and the close correlation of dietary FAs with pork IV (Wood et al., 2004; Benz et al., 2011; NRC, 2012; Kellner et al., 2016).

In the liver, as in adipose tissue, many FA composition changes were detected between the fat treatments. However, differences existed between the adipose tissues and liver in that C20:1, C20:4, and CLA that had no significant effect in the liver, and that C18:0 and C20:3 had a different directional response to the dietary fat sources from that observed in the adipose tissues. Additionally, C10:0 and C12:0 were not detected in liver while C22:2, C22:5, and C22:6 were present but were not detected in the backfat and belly fat of pigs. The fact that the liver had different FA profile from the adipose tissues agrees with our previous study (Wang et al., 2022b). Also in agreement with Wang et al. (2022b) is that C18:0, C:18:2n-6, and C20:4 were the most abundant FA in liver while C16:0 and C18:1 were the most abundant in the adipose tissues indicating that liver is the organ where the active FA metabolism and modification occurs (Jump et al., 2005; Duran-Montgé et al., 2009). The only interaction between dietary fat source and VE supplementation level in liver FA was observed for C16:1 in the current study. The interaction is not explainable and the general lack of interactions suggests that there may not be a notable dependence between fat source and VE supplementation level in tissue FA profile of pigs, which agrees with our previous study (Wang et al., 2022b).

With increasing dietary VE supplementation levels, there were slight increases observed in C20:0 and SFA in backfat, C18:0 and C18:3n-6 in belly fat, and C22:5 in liver while there were slight decreases observed in C16:1 and CLA in belly fat, and C22:0 in liver. Except for C18:0 in belly fat, the magnitude of change in these FA are very small, with some only noted as tendencies at 0.05 < *P* < 0.10, compared with major FA change in tissues related to the fat source. Therefore, the current results indicate that increasing dietary VE supplementation level has very limited potential to affect the FA profile of adipose tissues and liver. However, for muscle, Guo et al., (2006) reported that VE supplementation to the finishing pig diet could increase the percentage of UFA of neutral lipid fraction in loin muscle and reduce that of SFA. This response in muscle tissue, suggests that further studies are needed to demonstrate its effect clearly with regard to potential differential effects of VE on the FA profile in various tissues.

In the current study, α-T concentration in plasma increased with increasing dietary VE supplementation level from 11 to 200 ppm, which agrees with previous studies with pigs (Monahan et al., 1989; Corino et al., 1999; Niculita et al., 2007; Boler et al., 2009), with the greatest numerical increase when dietary VE supplementation level increased from 11 ppm up to 100 ppm. In the current study, the increase in plasma VE concentration with time slowed down after phase 3 (around 64 d), which agrees with Corino et al. (1999) that reported that the effect of increasing dietary VE supplementation level peaked at day 40 of feeding diets with supplemental VE. The increased intake of dietary VE was absorbed and deposited into the liver and muscle readily, and the deposition increased linearly with increasing dietary VE supplementation level. The result is in agreement with previous studies (Yang et al., 2009; Lauridsen et al., 2013). Corino et al (1999) also reported that increased dietary VE supplementation level increased loin muscle α-T concentrations in pigs.

Regarding the fat source effect, the α-T and total T concentrations in plasma at the end of phase 3 and in tissues (liver and loin muscle) upon slaughter decreased when the pigs were fed the DCO diet compared with the pigs fed the TW diet. Also, there was a greater increase of loin muscle α-T concentrations in the TW treatment than the DCO treatment with the increasing dietary VE level. High PUFA diets may result in lower tocopherol concentrations in plasma and tissues of pigs; it has previously been reported that high SFA content in the diet increases plasma and tissue tocopherol concentrations (Prévéraud et al., 2014; Wang et al., 2022b). Prévéraud et al. (2015) also reported a positive correlation between dietary MUFA content, mainly with oleic acid (C18:1), and tissue VE concentrations. Regarding the difference in the FA profile of different fat sources, VE emulsified in PUFA showed lower gastrointestinal absorption compared with that solubilized in MUFA (Gallo-Torres et al., 1977), which may potentially affect VE deposition in the body. Wang et al (2022b) reported that plasma α-tocopherol concentrations were greater in the pigs fed the TW diet compared with the pigs fed the DCO diet when 200 ppm of VE was supplemented to the diet. Therefore, the TW source could be more beneficial to enhance VE absorption and deposition in pigs compared with the DCO source.

In the current study, increasing dietary VE from 11 to 200 ppm quadratically increased SOD activity (highest at 100 ppm), and quadratically decreased MDA content (an end product of lipid oxidation; lowest at 40 to 100 ppm) in the liver. The result is in agreement with Lauridsen et al. (1999) that reported an improved enzymatic antioxidant system including SOD, catalase, and glutathione peroxidase with increasing VE supplementation levels.

In the evaluation of vaccine effectiveness, pigs were all negative in influenza antibodies at day 0, established their primary response to influenza at day 21, and gained full protection in the secondary response 14 d after the second vaccination (day 36). However, as with many immune measures, the variation was large which resulted in no clear dietary treatment effects on the primary measure of interest—the secondary response. Increased plasma tocopherol concentration was also reported to display a regulatory effect on leukocyte recruitment in mice, which was different between α-T and γ-T, can be affected by their concentration in the tissues, and was accompanied by a modest change in cytokines and chemokines (McCary et al., 2011). In this study, there were no notable significant differences in titers of different antibodies. Therefore, further measurements regarding leukocyte recruitment and regulatory cytokines may be helpful to better interpret the supplemental effect of the different isoforms of tocopherols.

In conclusion, the dietary FA composition of TW and DCO affected much of the FA profile in backfat, belly fat, and liver but increasing VE supplementation level did not materially alter the FA profile in these tissues. Increasing dietary VE supplementation level increased total tocopherol concentrations (mainly α-T) in blood and tissues and improved liver antioxidant capacity. With regard to interactions between VE supplementation level and DCO and TW as fat sources, the TW in the diet increased both absorption and deposition of tocopherol greater than the DCO with increasing dietary VE supplementation levels.

## References

[CIT0001] Apple, J. K., C. Maxwell, D. Galloway, C. Hamilton, and J. Yancey. 2009a. Interactive effects of dietary fat source and slaughter weight in growing-finishing swine: II. Fatty acid composition of subcutaneous fat. J. Anim. Sci. 87:1423–1440. doi:10.2527/jas.2008-1454. 19066245

[CIT0002] Apple, J. K., C. Maxwell, D. Galloway, C. Hamilton, and J. Yancey. 2009b. Interactive effects of dietary fat source and slaughter weight in growing-finishing swine: III. Carcass and fatty acid compositions. J. Anim. Sci. 87:1441–1454. doi:10.2527/jas.2008-1455. 19066247

[CIT0004] Barnett, V., and T. Lewis. 1994. Outliers in statistical data. New York. NY: John Wiley & Sons.

[CIT0005] Benz, J. M., M. D. Tokach, S. S. Dritz, J. L. Nelssen, J. M. DeRouchey, R. C. Sulabo, and R. D. Goodband. 2011. Effects of choice white grease and soybean oil on growth performance, carcass characteristics, and carcass fat quality of growing-finishing pigs. J. Anim. Sci. 89:404–413. doi:10.2527/jas.2009-2737. 21262976

[CIT0006] Blatt, D. H., W. A. Pryor, J. E. Mata, and R. Rodriguez-Proteau. 2004. Re-evaluation of the relative potency of synthetic and natural α-tocopherol: experimental and clinical observations. J. Nutr. Biochem. 15:380–395. doi:10.1016/j.jnutbio.2003.12.011. 15219923

[CIT0007] Boler, D. D., S. R. Gabriel, H. Yang, R. Balsbaugh, D. C. Mahan, M. S. Brewer, F. K. McKeith, and J. Killefer. 2009. Effect of different dietary levels of natural-source vitamin E in grow-finish pigs on pork quality and shelf life. Meat Sci. 83:723–730. doi:10.1016/j.meatsci.2009.08.012. 20416631

[CIT0008] Corino, C., G. Oriani, L. Pantaleo, G. Pastorelli, and G. Salvatori. 1999. Influence of dietary vitamin E supplementation on “heavy” pig carcass characteristics, meat quality, and vitamin E status. J. Anim. Sci. 77:1755–1761. doi:10.2527/1999.7771755x. 10438022

[CIT0009] de Tonnac, A., M. Guillevic, and J. Mourot. 2018. Fatty acid composition of several muscles and adipose tissues of pigs fed n-3 PUFA rich diets. Meat Sci. 140:1–8. doi:10.1016/j.meatsci.2017.11.023. 29477879

[CIT0010] Duran-Montgé, P., P. K. Theil, C. Lauridsen, and E. Esteve-Garcia. 2009. Dietary fat source affects metabolism of fatty acids in pigs as evaluated by altered expression of lipogenic genes in liver and adipose tissues. Animal. 3:535–542. doi:10.1017/S1751731108003686. 22444377

[CIT0011] Gallo-Torres, H., J. Ludorf, and M. Brin. 1977. The effect of medium-chain triglycerides on the bioavailability of vitamin E. Int. J. Vitam. Nutr. Res. 48:240–241. 711382

[CIT0012] Guo, Q., B. T. Richert, J. R. Burgess, D. M. Webel, D. E. Orr, M. Blair, A. L. Grant, and D. E. Gerrard. 2006. Effect of dietary vitamin E supplementation and feeding period on pork quality. J. Anim. Sci. 84:3071–3078. doi:10.2527/jas.2005-578. 17032801

[CIT0013] Hasty, J. L., E. van Heugten, M. T. See, and D. K. Larick. 2002. Effect of vitamin E on improving fresh pork quality in Berkshire- and Hampshire-sired pigs. J. Anim. Sci. 80:3230–3237. doi:10.2527/2002.80123230x. 12542164

[CIT0014] Jensen, M. S., S. K. Jensen, and K. Jakobsen. 1997. Development of digestive enzymes in pigs with emphasis on lipolytic activity in the stomach and pancreas. J. Anim. Sci. 75:437–445. doi:10.2527/1997.752437x. 9051467

[CIT0015] Jump, D. B., D. Botolin, Y. Wang, J. Xu, B. Christian, and O. Demeure. 2005. Fatty acid regulation of hepatic gene transcription. J. Nutr. 135:2503–2506. doi:10.1093/jn/135.11.2503. 16251601

[CIT0016] Kellner, T., N. Gabler, and J. Patience. 2017. The composition of dietary fat alters the transcriptional profile of pathways associated with lipid metabolism in the liver and adipose tissue in the pig. J. Anim. Sci. 95:3609–3619. doi:10.2527/jas.2017.1658. 28805896

[CIT0017] Kellner, T., G. Gourley, S. Wisdom, and J. Patience. 2016. Prediction of porcine carcass iodine value based on diet composition and fatty acid intake. J. Anim. Sci. 94:5248–5261. doi:10.2527/jas.2016-0643. 28046146

[CIT0018] Kloareg, M., J. Noblet, and J. van Milgen. 2007. Deposition of dietary fatty acids, de novo synthesis and anatomical partitioning of fatty acids in finishing pigs. Br. J. Nutr. 97:35–44. doi:10.1017/S0007114507205793. 17217558

[CIT0019] Lauridsen, C., J. H. Nielsen, P. Henckel, and M. T. Sørensen. 1999. Antioxidative and oxidative status in muscles of pigs fed rapeseed oil, vitamin E, and copper. J. Anim. Sci. 77:105–115. doi:10.2527/1999.771105x. 10064033

[CIT0020] Lauridsen, C., P. K. Theil, and S. K. Jensen. 2013. Composition of α-tocopherol and fatty acids in porcine tissues after dietary supplementation with vitamin E and different fat sources. Anim. Feed Sci. Technol. 179:93–102. doi:10.1016/j.anifeedsci.2012.10.007.

[CIT0021] McCary, C. A., H. Abdala-Valencia, S. Berdnikovs, and J. M. Cook-Mills. 2011. Supplemental and highly elevated tocopherol doses differentially regulate allergic inflammation: reversibility of α-tocopherol and γ-tocopherol’s effects. J. Immunol. 186:3674–3685. doi:10.4049/jimmunol.1003037. 21317387 PMC3271805

[CIT0022] Meadus, W. J., P. Duff, B. Uttaro, J. L. Aalhus, D. C. Rolland, L. L. Gibson, and M. E. R. Dugan. 2010. Production of docosahexaenoic acid (DHA) enriched bacon. J. Agric. Food Chem. 58:465–472. doi:10.1021/jf9028078. 19924860

[CIT0023] Mitchaothai, J., C. Yuangklang, S. Wittayakun, K. Vasupen, S. Wongsutthavas, P. Srenanul, R. Hovenier, H. Everts, and A. C. Beynen. 2007. Effect of dietary fat type on meat quality and fatty acid composition of various tissues in growing–finishing swine. Meat Sci. 76:95–101. doi:10.1016/j.meatsci.2006.10.017. 22064195

[CIT0024] Monahan, F., D. Buckley, P. Morrissey, P. Lynch, and J. Gray. 1989. Effect of dietary α-tocopherol supplementation on α-tocopherol levels in porcine tissues and on susceptibility to lipid peroxidation. Food Sci. Nutr. 42:203–212. doi:10.1080/09543465.1989.11904145.

[CIT0025] Niculita, P., M. E. Popa, M. Ghidurus, and M. Turtoi. 2007. Effect of vitamin E in swine diet on animal growth performance and meat quality parameters. Polish J. Food Nutr. Sci. 57:125–130.

[CIT0026] NRC. 2012. Nutrient requirements of swine. 11th ed. Washington, DC, USA: National Academies Press.

[CIT0027] Nuernberg, K., K. Fischer, G. Nuernberg, U. Kuechenmeister, D. Klosowska, G. Eliminowska-Wenda, I. Fiedler, K. Ender. 2005. Effects of dietary olive and linseed oil on lipid composition, meat quality, sensory characteristics and muscle structure in pigs. Meat Sci. 70:63–74. doi:10.1016/j.meatsci.2004.12.001. 22063281

[CIT0028] Onibi, G. E., J. R. Scaife, I. Murray, and V. R. Fowler. 2000. Supplementary α-tocopherol acetate in full-fat rapeseed-based diets for pigs: effect on performance, plasma enzymes and meat drip loss. J. Sci. Food Agric. 80:1617–1624. doi:10.1002/1097-0010(20000901)80:11<1617::aid-jsfa689>3.0.co;2-3.

[CIT0029] Park, P., and R. Goins. 1994. In situ preparation of fatty acid methyl esters for analysis of fatty acid composition in foods. J. Food Sci. 59:1262–1266. doi:10.1111/j.1365-2621.1994.tb14691.x.

[CIT0030] Prévéraud, D., C. Desmarchelier, F. Rouffineau, E. Devillard, and P. Borel. 2015. A meta-analysis to assess the effect of the ­composition of dietary fat on α-tocopherol blood and tissue concentration in pigs. J. Anim. Sci. 93:1177–1186. doi:10.2527/jas.2014-8422. 26020895

[CIT0031] Prévéraud, D. P., E. Devillard, F. Rouffineau, and P. Borel. 2014. Effect of the type of dietary triacylglycerol fatty acids on α-tocopherol concentration in plasma and tissues of growing pigs. J. Anim. Sci. 92:4972–4980. doi:10.2527/jas.2013-7099. 25349346

[CIT0032] Ramsay, T., C. Evock-Clover, N. Steele, and M. Azain. 2001. Dietary conjugated linoleic acid alters fatty acid composition of pig skeletal muscle and fat. J. Anim. Sci. 79:2152–2161. doi:10.2527/2001.7982152x. 11518224

[CIT0033] Rentfrow, G., T. Sauber, G. Allee, and E. Berg. 2003. The influence of diets containing either conventional corn, conventional corn with choice white grease, high oil corn, or high oil high oleic corn on belly/bacon quality. Meat Sci. 64:459–466. doi:10.1016/s0309-1740(02)00215-2. 22063128

[CIT0034] Świątkiewicz, M., A. Olszewska, E. R. Grela, and M. Tyra. 2021. The effect of replacement of soybean meal with corn dried distillers grains with solubles (cDDGS) and differentiation of dietary fat sources on pig meat quality and fatty acid profile. Animals. 11:1277. doi:10.3390/ani11051277. 33946686 PMC8146195

[CIT0035] Trefan, L., L. Bünger, J. Bloom-Hansen, J. A. Rooke, B. Salmi, C. Larzul, C. Terlouw, and A. Doeschl-Wilson. 2011. Meta-analysis of the effects of dietary vitamin E supplementation on α-tocopherol concentration and lipid oxidation in pork. Meat Sci. 87:305–314. doi:10.1016/j.meatsci.2010.11.002. 21146329

[CIT0041] Tse, M., M. Kim, C. H. Chan, P. L. Ho, S. Ma, Y. Guan, and J. S. M. Peiris. 2012. Evaluation of three commercially available influenza A type-specific blocking enzyme-linked immunosorbent assays for seroepidemiological studies of influenza A virus infection in pigs. Clinical and Vaccine Immunology 19: 334-337. doi:10.1128/CVI.05358-11. 22219314 PMC3294623

[CIT0036] Wang, D., Y. D. Jang, G. K. Rentfrow, M. J. Azain, and M. D. Lindemann. 2022a. Effects of dietary vitamin E and fat supplementation in growing-finishing swine fed to a heavy slaughter weight of 150 kg: I. Growth performance, lean growth, organ size, carcass characteristics, primal cuts, and pork quality. J. Anim. Sci. 100:1–16. doi:10.1093/jas/skac081. PMC9030125 35289901

[CIT0037] Wang, D., Y. D. Jang, G. K. Rentfrow, M. J. Azain, and M. D. Lindemann. 2022b. Effects of dietary vitamin E and fat supplementation in growing-finishing swine fed to a heavy slaughter weight of 150 kg: II. Tissue fatty acid profile, vitamin E concentrations and antioxidant capacity of plasma and tissue. J. Anim. Sci. 100:1–13. doi:10.1093/jas/skac184. PMC9182394 35584810

[CIT0038] Wang, D., Y. D. Jang, G. K. Rentfrow, M. J. Azain, and M. D. Lindemann. 2023. Effects of dietary fat source and multiple ­vitamin E levels in growing-finishing swine fed to heavy slaughter weight of 150 kg: I. Growth performance, lean growth, organ size, carcass characteristics, primal cuts, and pork quality. T. Anim. Sci. txad086, doi:10.1093/tas/txad086. PMC9030125 35289901

[CIT0039] Wood, J. D., R. I. Richardson, G. R. Nute, A. V. Fisher, M. M. Campo, E. Kasapidou, P. R. Sheard, and M. Enser. 2004. Effects of fatty acids on meat quality: a review. Meat Sci. 66:21–32. doi:10.1016/S0309-1740(03)00022-6. 22063928

[CIT0040] Yang, H., D. C. Mahan, D. A. Hill, T. E. Shipp, T. R. Radke, and M. J. Cecava. 2009. Effect of vitamin E source, natural versus synthetic, and quantity on serum and tissue α-tocopherol concentrations in finishing swine. J. Anim. Sci. 87:4057–4063. doi:10.2527/jas.2008-1570. 19717777

